# Integrative disease classification based on cross-platform microarray data

**DOI:** 10.1186/1471-2105-10-S1-S25

**Published:** 2009-01-30

**Authors:** Chun-Chi Liu, Jianjun Hu, Mrinal Kalakrishnan, Haiyan Huang, Xianghong Jasmine Zhou

**Affiliations:** 1 Molecular and Computational Biology, University of Southern California, CA, USA; 2 Department of Statistics, University of California, Berkeley, CA, USA

## Abstract

**Background:**

Disease classification has been an important application of microarray technology. However, most microarray-based classifiers can only handle data generated within the same study, since microarray data generated by different laboratories or with different platforms can not be compared directly due to systematic variations. This issue has severely limited the practical use of microarray-based disease classification.

**Results:**

In this study, we tested the feasibility of disease classification by integrating the large amount of heterogeneous microarray datasets from the public microarray repositories. Cross-platform data compatibility is created by deriving expression log-rank ratios within datasets. One may then compare vectors of log-rank ratios across datasets. In addition, we systematically map textual annotations of datasets to concepts in Unified Medical Language System (UMLS), permitting quantitative analysis of the phenotype "distance" between datasets and automated construction of disease classes. We design a new classification approach named ManiSVM, which integrates Manifold data transformation with SVM learning to exploit the data properties. Using the leave one dataset out cross validation, ManiSVM achieved the overall accuracy of 70.7% (68.6% precision and 76.9% recall) with many disease classes achieving the accuracy higher than 80%.

**Conclusion:**

Our results not only demonstrated the feasibility of the integrated disease classification approach, but also showed that the classification accuracy increases with the number of homogenous training datasets. Thus, the power of the integrative approach will increase with the continuous accumulation of microarray data in public repositories. Our study shows that automated disease diagnosis can be an important and promising application of the enormous amount of costly to generate, yet freely available, public microarray data.

## Background

Microarray technology provides a revolutionary tool for understanding human diseases. Golub et al. [1] demonstrated that microarray data can be used to classify cancer, e.g. to distinguish between acute myeloid leukemia and acute lymphocytic leukemia. Since then, disease classification has been one of the primary foci of microarray research. For example, microarray technology has been applied to classify cancers as diverse as lung cancer [2], breast cancer [3], and glioma [4]. In principle, a disease classification problem can be solved with a two-step process: (1) build classifiers based on samples with known disease class labels; and (2) classify the unknown samples into known disease classes. In an ideal case, we would hope that the large amount of data generated by different laboratory on various diseases could be integrated into a diagnosis database, such that unknown samples could then be matched to the disease classes in the database. In this way, microarray-based classification could be practical and promising.

Recently, several studies have tested the feasibility of disease classification on cross-platform microarray data [5-9]. Employing different normalization methods, those studies showed promising results. However, all of those studies were based on cancer microarray data with limited scales. Moreover, in some studies, the good performance was biased by correlated training and testing data (samples from the same dataset were distributed into training and testing data) [5,7]. In addition, the performance evaluations of current studies were mainly focused on precision without considering recall. In this study, we integrated 68 microarray datasets of diverse disease classes to perform a large-scale and unbiased evaluation on the classification performance. Furthermore, we design an approach to automatically construct disease classes from microarray data, which is an important step towards automated disease classification by utilizing the enormous amount of public microarray repositories.

Our goal is that given microarray data profiling two samples, one normal condition and another disease condition, the disease condition can be classified based on the phenotype annotations of datasets in the public microarray database. To approach this problem, we need three component tools: (1) a feature vector to describe a microarray profile pair (disease vs. normal) that is comparable among microarray data generated with different platforms; (2) disease classes built from cross-platform microarray data based on their associated phenotype information; and (3) a machine learning approach capable of assigning potential phenotypes to a queried sample pair based on its similarity to profiled pairs in known disease classes.

For the first component, we derive the expression log-rank ratio for each gene in each profile pair. By first deriving the expression log-rank ratios between a disease and a normal profile as meta-information within the same dataset, and then comparing such ratio profiles across datasets, the results shall be comparable across datasets. Simply speaking, we compare cross-dataset signals by emphasizing on differentially expressed genes, which were shown to be relatively robust to platforms or laboratory settings[10]. To complete the second component, we need to systematically annotate the experimental information associated with each microarray dataset. We followed the approach of Butte and Kohane [11] to use the disease concepts in the Unified Medical Language Systems (UMLS) [12] in order to annotate the phenotypes associated with each microarray dataset. Since a disease state is usually defined by several phenotype concepts (e.g. cancer, liver tissue, metastasis), we built disease classes by selecting microarray datasets sharing a common set of UMLS concepts. With respect to the third component, we used Support Vector Machine (SVM) [13,14] for classification, and further developed a method named ManiSVM by integrating Manifold [15] and SVM where Manifold is employed for nonlinear dimensionality reduction to enhance the performance.

By integrating the microarray data of major platforms in the NCBI Gene Expression Omnibus (GEO) database [16], we constructed 117 classes. Using the leave one dataset out cross validation (LOOCV), ManiSVM and SVM achieved the overall accuracies of 70.7% and 58.8%, respectively. Our result not only demonstrates the feasibility of disease diagnosis by integrating heterogeneous microarray data, but also reveals that the performance of disease classification improves with the number of homogenous training datasets. Thus, the power of the integrative approach can be expected to dramatically increase with the continued accumulation of microarray data in public repositories.

## Results

**Figure 1:**
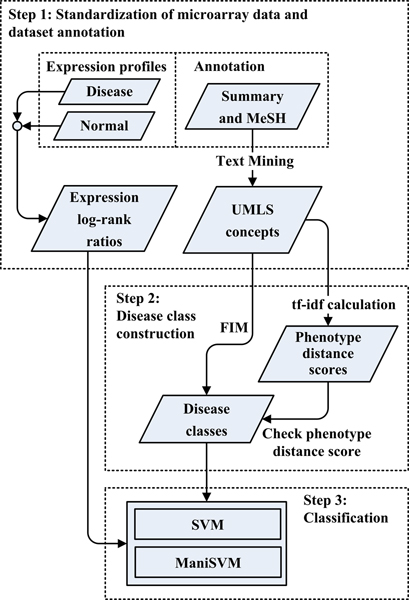
**Diagram of the integrative disease classification framework**. The framework consists of three major steps: (**1**) Standardization of microarray data and dataset annotation: expression log-rank-ratio vectors were constructed from each microarray data set, and UMLS concepts were extracted from the dataset summary and corresponding MeSH headings. (**2**) Disease class construction: disease classes were initially constructed by FIM analysis and were further refined by calculating the *phenotype distance score*. (**3**) Classification by SVM and ManiSVM.

In this section, we will first give a brief introduction to the methods, followed by analysis results. Figure 1 illustrates the three major steps of our analysis: (1) data standardization, (2) construction of disease classes, and (3) classification.

### Data standardization for cross-dataset comparison

#### Standardization of expression data

We collected 232 human microarray datasets from three major platforms of the NCBI GEO [16]: U95, U133, and U133 plus 2.0. These platforms contain the majority of GEO human datasets. We only kept the 80 datasets containing both diseased and "normal" (or "control") conditions. The mapping between probe sets and Entrez Gene ID [17] yielded a set of 8229 genes common to all three platforms. For each gene, we calculate the average expression level for probe sets associated to this gene. Within each dataset, any combination of one disease sample and one normal sample is called a profile pair. To avoid systematic bias due to the differences in expression signals measured by different laboratories, we describe the properties of a profile pair in terms of log-rank ratios. This is calculated as follows: (1) convert the gene expression values to ranks within each profile, and (2) calculate the log ratio of the two ranks for each gene (disease rank/normal rank). In total, we obtained 12,802 vectors of log-rank ratios for an equivalent number of profile pairs. We then filtered out those datasets giving rise to fewer than 5 such vectors. Our final sample consists of 68 datasets and 12,767 log-rank-ratio vectors.

#### Standardization of dataset annotation

To systematically categorize the phenotype information associated with each microarray dataset, we mapped the MeSH headings and the GEO dataset summary of each dataset to the UMLS concepts. Any UMLS concepts associated with only one microarray dataset were filtered out, resulting in a vocabulary of 185 disease and phenotype concepts. More details on this phase of the analysis are given in the Methods section.

### Construction of disease classes

This step groups microarray datasets into disease classes. We first employed the frequent itemset mining (FIM) algorithm [18] to identify candidate disease groups sharing a common set of UMLS concepts. This effort assumes that a particular disease state is usually described by a common group of UMLS concepts (for example, all "breast cancer" datasets match the UMLS concepts "breast" and "neoplasms"). Next, within each group we measured the phenotype distance score among pairs of datasets. This is quantified by the *term frequency-inverse document frequency *(*tf-idf*) [19]. Only those disease classes with a *phenotype distance score *whose estimated *p*-value is less than 0.05 were kept for further analysis. This cut ensures that each class has a similar level of homogeneity in its associated UMLS concepts. Details of the *tf-idf *calculation and its associated *p*-value estimation are described in the Methods section.

In this manner we constructed 117 classes, comprising 68 microarray datasets. Each class contains 3 to 12 datasets. The classes covered a wide spectrum of conditions: cardiovascular/heart diseases, "bacterial infections and mycoses", neoplasms, CNS disorders, skin disorders, and metabolic diseases. Table 1 shows selected disease classes. Note that a given dataset can appear in more than one class, and that many of the classes are interrelated. For example, the disease class described by (neoplasms, "neoplasms, glandular and epithelial", and "neoplasms by histologic type") is the parent class of one characterized by (carcinoma, neoplasms, "neoplasms, glandular and epithelial", and "neoplasms by histologic type").

**Table 1:** Selected disease classes and their associated classification performance.

UMLS concepts	Datasets	Phenotype distance score (*p*-value)	ManiSVM accuracy	SVM accuracy
C0027651 (Neoplasms), C0027660 (Neoplasms, Glandular and Epithelial), C0040300 (Body tissue), C0007097 (Carcinoma), C0027653 (Neoplasms by Site), C0027652 (Neoplasms by Histologic Type)	GDS1070 GDS1321 GDS1479 GDS505	3.50E-05	0.8421	0.6018

C0018981 (Hemic and Lymphatic Diseases), C0005773 (Blood Cells), C0018939 (Hematological Disease)	GDS1257 GDS1392 GDS539 GDS1320 GDS390	7.10E-05	0.8047	0.6253

C0007682 (CNS disorder), C0006111 (Brain Diseases), C0027765 (nervous system disorder)	GDS1331 GDS1726 GDS1065	9.99E-03	0.7569	0.6483

C0021311 (Infection), C0004615 (Bacterial Infections and Mycoses)	GDS1428 GDS1022 GDS539 GDS711 GDS1726 GDS1397	2.36E-04	0.7498	0.5253

The datasets within each disease class naturally form a *positive set *for that class. However, the size of the datasets can vary widely. Large datasets may come to dominate the characteristics of their disease classes. In order to get an unbiased estimator of classification accuracy, we randomly selected 50 log-rank ratio vectors from each dataset if its total number of profile pairs is greater than 50. We built the *negative set *by randomly sampling an equal number of vectors from datasets not in the *positive set*.

### Classification analysis

We applied two approaches to training the disease classifiers. The first was direct application of the SVM algorithm with a linear kernel and C-support vector classification (C-SVC), using the LIBSVM package [20]. Prior to classification, we reduced the number of features by selecting those genes with significantly different log-rank ratios (*t*-test *p*-value < 0.05) between the positive and negative training sets. In the second approach, we first constructed a Laplacian matrix to represent the gene expression data [15], thereby transforming the data in a non-linear fashion into a new and lower-dimensional manifold. We then applied SVM to the transformed data. We call the second approach ManiSVM. The major advantage in integrating graph laplacian with SVM is to transform the data via non-linear dimension reduction into a new space, where data points close in distance shall share high phenotype similarity based on the chosen similarity metric. Such transformation enhances the separation of data points between positive and negative classes, thus the subsequent application of the linear kernel SVM to the transformed data can achieve better performance than its direct application to the original data. Details of the graph laplacian transformation are described in Methods.

We performed disease classification with SVM and ManiSVM, and evaluated performance with LOOCV (see Methods for details). We performed LOOCV by leaving out one dataset from the positive set (within a disease class) and the equal number of expression log-rank-ratio vectors from the negative set as the testing positive and negative set, respectively. Then the remainder positive and negative set are training positive and negative set, respectively. Even though our classification unit is a single profile, we left out the entire positive dataset to avoid bias caused by replicates in the same dataset. We used the following measures to assess classification performance: precision = TP/(TP+FP); recall = TP/(TP+FN); and accuracy = (TP+TN)/(TP+TN+FP+FN), where TP stands for true positives, TN for true negatives, FP for false positives, and FN for false negatives. The accuracy is actually a way to summarize the precision and recall information. For each LOOCV procedure, we repeated 5 times by random sampling of different negative sets, and we averaged the result to assess the classification performance.

Our results showed that ManiSVM achieved the overall accuracy of 70.7% outperforming SVM (58.8%) by the default hyperplane positions. Although SVM can achieve high classification precision (89.8%), its recall is rather low (19.8%). In the contrast, ManiSVM provides more balanced performance and yielded 68.6% precision and 76.9% recall with 12% disease classes achieving the accuracy higher than 80%. By further shifting the position of hyperplane via adjusting the threshold of SVM decision value, SVM achieved the maximum accuracy of 67.5% (72.0% precision and 57.3% recall) and ManiSVM achieved the maximum accuracy of 75.6% (68.6% precision and 94.4% recall). Again, ManiSVM outperformed SVM. The endowed classification power of ManiSVM is attributed to the fact that the data points can be better separated in the Manifold space in terms of their phenotype similarity based on the chosen data similarity metric.

**Figure 2:**
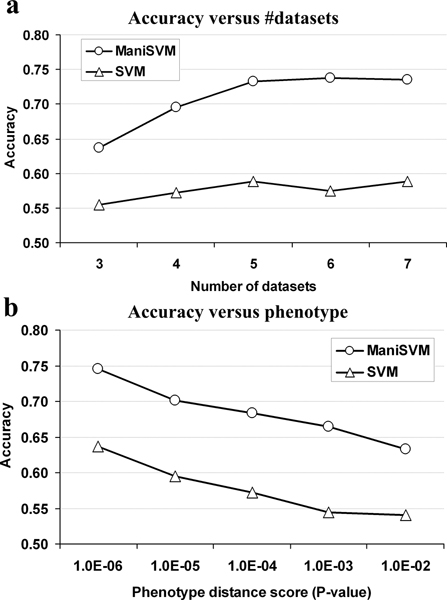
**Classification performance increases with size and phenotype homogeneity of disease classes**. The disease classes were divided into bins (**a**) based on the number of datasets (from 3 to 7) in the classes, or (**b**) based on the *p*-value of the phenotype distance score (*p*-value intervals were chosen as: 1.0E-6 to 1.0E-5, 1.0E-5 to 1.0E-4, 1.0E-4 to 1.0E-3, 1.0E-3 to 1.0E-2, and 1.0E-2 to 5.0E-2). For each bin, the average accuracy was calculated by performing ManiSVM and SVM classification.

The performance of individual classes varies greatly. For example, ManiSVM achieved 83.4% accuracy for the disease class described by the UMLS concepts (neoplasms, neoplasms by site, and neoplasms by histologic type), but only 67.2% accuracy for the disease class described by (neoplasms, mammary neoplasms, and neoplasms by site). One reason for this difference is that the former contains 9 datasets, while the latter contains only 3 datasets. In general, a class with more datasets will be easier to classify (or more properly, will have more capacity to train a classifier). Figure 2a shows that the accuracy increases with the number of datasets in a class: as the number of datasets increases from 3 to 7, the accuracy increases from 63.7% to 73.5% and from 55.4% to 58.8% for ManiSVM and SVM respectively. This relationship highlights the advantage of integrating multiple datasets for disease classification.

## Discussion

Although the correlation between dataset number and classification performance is strong (Figure 2a), outliers do exist. For example, the disease class characterized by the UMLS concepts (neoplasms, "neoplasms, glandular and epithelial", carcinoma, "head and neck neoplasms", neoplasms by site, and neoplasms by histologic type) contains only 3 datasets, but has a high classification accuracy of 82.8%. In contrast, the disease class (leukocytes, immune system diseases, and blood cells) contains 4 datasets, but is associated with a classification accuracy of 66.0%. These two disease classes differ in terms of within-class homogeneity. The disease class with 3 datasets benefited from similar dataset annotations, with an average phenotype distance score of 0.59; while the disease class with 4 datasets had an average phenotype distance score of 0.78.

To properly compare the average phenotype distances within disease classes of different sizes (3-12 datasets), we estimated the statistical significance of phenotype distance scores by random sampling (see the Methods section). In general, more significant *p*-values correspond to lower phenotype distance scores and higher degrees of within-class data homogeneity. Figure 2b shows the significant negative correlation between accuracy and the *p*-value of the phenotype distance score. As the *p*-value of the phenotype distance score increases from 10^-6 ^to 10^-2^, the accuracy of the classifier decreases from 74.6% to 63.3% and from 63.7% to 54.0% for ManiSVM and SVM respectively. This analysis demonstrates conclusively that classification power increases with dataset homogeneity. Thus, integrating multiple datasets is only expected to enhance classification performance if they are sufficiently homogenous in the diseases being measured.

In the above analysis, we have assessed the homogeneity of a disease class by comparing dataset annotations that were mapped to the UMLS concepts. Our results show that such assessment generally reflects the true phenotype similarity between datasets. Despite a satisfactory overall performance, we have observed a few exceptions to this rule. For example, the disease class (digestive system disorders and epithelial cells) contained 3 datasets {GDS858, GDS1321, GDS1022} and had a fairly small average phenotype distance score 0.67 (*p*-value 0.008), but proved rather difficult to classify. A further investigation of individual datasets revealed that the dataset GDS1022 actually studied lung pneumocytes after infection with Pseudomonas aeruginosa, which is not related to digestive system disorders. The reason that GDS1022 was mapped to this disease class is because its dataset summary mentioned "Pseudomonas aeruginosa causes serious respiratory infections in cystic fibrosis patients," and "cystic fibrosis" was automatically mapped to the "digestive system disorders" by the UMLS system. Thus, although the object of this study (lung pneumocytes) is *not *related to the digestive system, GDS1022 was nonetheless classed among digestive system disorders by the UMLS concept of "cystic fibrosis." This mapping imprecision resulted in inaccurate phenotype distance scores, and led to a low classification accuracy of 60.2% and 59.1% for this disease class by ManiSVM and SVM, respectively. On the other hand, such failures prove that the performance of our method could be further enhanced by a more advanced UMLS text mining tool. As with all automated text mining methods, however, mapping imprecision cannot be fully avoided. But with the rapid accumulation of microarray data, we should be able to minimize or bypass the influence of UMLS mapping noise by imposing stricter homogeneity requirements on candidate disease classes.

## Conclusion

We have proposed a framework for microarray-based molecular diagnosis by combining public microarray repositories with the UMLS knowledge base. We respond to several challenges in integrating cross-platform microarray datasets. In particular, we addressed the issue of data compatibility by expressing the difference in two profiles as the ratio of logarithmic rankings. In addition, we systematically associated each microarray dataset with disease classes by mapping their textual annotations to UMLS concepts. The disease classes were created by comparing phenotype distance scores among pairs of datasets. Although SVM has already been considered one of the best approaches for microarray-based disease classification by several studies [21,22], we further enhanced its power by using Manifold for non-linear dimension reduction and data transformation. Our result has not only demonstrated the feasibility of this approach, but also highlighted the fact that classification power increases with the number and homogeneity of training datasets. This work therefore provides a solid foundation to the problem of integrating enormous amounts of microarray data, which are costly to generate yet freely available. The power of our approach will increase dramatically with the continued growth of public microarray repositories. The framework presented here will also benefit from ongoing efforts to develop more advanced UMLS text mining tools.

## Methods

### Disease annotation with UMLS concepts

To systematically categorize the phenotypes associated with each microarray dataset, we used the UMLS system [12,23]. For each dataset, we identified its associated publication and downloaded its medical subject headings (MeSH) via NCBI Entrez programming utilities. The MeSH and NCBI GEO summary of a dataset were then parsed with the program MetaMap to find UMLS concepts. To reduce noise we focused on a subset of disease-related concepts in UMLS, including all the MeSH vocabulary and terms belonging to the semantic types: pathologic function, "injury or poisoning", anatomical abnormality, "body part, organ, or organ component", tissue, and cell. To infer higher-order links between datasets, all ancestor concepts were included.

### Calculation of the phenotype distance scores and the associated p-values

The calculation procedure is as follows:

1. For the UMLS concept *i *in dataset *j*, we calculated the *term frequency tf*(*i*, *j*) = *n*_*i*, *j*_/(∑_*k *_*n*_*k*, *j*_), where *n*_*i*, *j *_denotes the number of occurrences of UMLS concept *i *in dataset *j*. Then by definition, the value of *tf*_(*i*, *j*) _indicates the level of occurrence frequency of UMLS concept *i *in dataset *j*.

2. We calculated the *inverse document frequency idf*_*i *_= log(*D*/*D*_*i*_), where *D *denotes the total number of datasets and *D*_*i *_is the number of datasets containing the UMLS concept *i*. A smaller *idf*_*i *_implies a higher popularity of UMLS concept *i *among the collected microarray datasets.

3. The *tf-idf *score was defined by *tf-idf*_(*i*, *j*) _= *tf*_(*i*, *j*) _× *idf*_*i*_, which adjusted the score of *tf*_(*i*, *j*) _by taking into account the popularity level of the UMLS concept *i*. More intuitively, *tf-idf*_(*i*, *j*) _can be considered as a measure of specific relevance of UMLS concept *i *to dataset *j*. Let *s *be the number of UMLS concepts, a dataset *j *is then associated with a *tf-idf *vector of dimension *s*, i.e., [*tf-idf*_(1, *j*)_,..., *tf-idf*_(*s*, *j*)_].

4. The phenotype similarity between any two datasets was estimated with the cosine between their *tf-idf *vectors. The *phenotype distance score *of a candidate disease class was calculated as one minus the average phenotype similarity of any dataset pair within the class.

5. Finally, to evaluate the significance of a *phenotype distance score*, we estimated its empirical *p*-value by bootstrapping all of the datasets. In detail, given a disease class with *k *datasets, we randomly sampled *k *datasets from all datasets, and calculated the *phenotype distance score*, repeated 1,000,000 times, and generated the empirical distribution.

### Graph laplacian transformation

In the following, we detail the graph laplacian transformation [14]. Given *k *expression log-rank-ratio vectors *x*_1_, *x*_2_,⋯,*x*_*k *_∈ ℜ^*l *^where *l *is the number of selected genes, we assume that the first *s *<*k *vectors are in the training set with labels *c*_*i*_, where *c*_*i *_= 1 if *x*_*i *_is in positive set and *c*_*i *_= -1 otherwise. The rest {*x*_*s*+1_, *x*_*s*+2_,⋯,*x*_*k*_} are in the testing set and unlabeled. The graph laplacian procedures are as follows:

1. Constructing the adjacency matrix: (1) calculate Pearson correlation for each vector pair *x*_*i *_and *x*_*j*_; (2) define adjacency matrix *W *as *w*_*ij *_= 1 if the Pearson correlation of the vector *x*_*i *_and *x*_*j *_is greater than the threshold *γ*; and *w*_*ij *_= 0 otherwise. Here we set *γ *to be 0.25.

2. Singular value decomposition (SVD): (1) build laplacian matrix *L *= *D *- *W *where *W *is the adjacency matrix defined above and *D *is a diagonal matrix of the same size as *W *satisfying *D*_*ij *_= ∑_*j *_*w*_*ji*_; (2) identify the eigenvalues and eigenvectors by solving the equation *L****e ***= *λ****e ***for *λ *and ***e***, and order the obtained *k *eigenvalues increasingly: *λ*_1 _≤ *λ*_2 _≤ ⋯ ≤*λ*_*k*_. The *p *eigenvectors *e*_1_, *e*_2_, ⋯ *e*_*p *_that corresponds to the *p *smallest eigenvalues *λ*_1_, *λ*_2_, ⋯, *λ*_*p *_are then used to represent *x*_1_, *x*_2_,⋯, *x*_*k *_in the manifold space, where $p=\underset{j=1,..,k}{\arg\max}\{\sum_{i=1}^{j}\lambda_{i};(\sum_{i=1}^{j}\lambda_{i})/(\sum_{i=1}^{k}\lambda_{i})<0.005\}$. That is that *x*_*i *_∈ ℜ^*l *^(*j *= 1,...,*k*) is mapped to (*e*_1_(*i*),...,*e*_*p*_(*i*)) with *e*_*j*_(*i*) being the *i*th component of the eigenvector ***e***_*j*_. We note that all the eigenvalues are non-negative since *L *is symmetric and positive semi-definite.

Following the notations in Belkin and Niyogi [15], we let *E*_*lab *_denote the *k *× *p *matrix with *e*_1_, *e*_2_, ⋯, *e*_*p *_being the column vectors of *E*_*lab *_(the (*i*, *j*)th entry of *E*_*lab *_is *e*_*j*_(*i*)). Then *E*_*lab *_represents the transformed data in the manifold space with a reduced dimension of *p*, as described above., SVM analysis is subsequently performed on these transformed data. The motivation for performing the manifold transformation comes from the following important mathematical property of *E*_*lab*_:

For any linear operation on *E*_*lab*_, say $(c_{1}^{*},...,c_{k}^{*})=(f_{1},...,f_{p})E_{lab}^{T}$ with $c_{i}^{*}$ = (*f*_1_,...,*f*_*p*_)(*e*_1_(*i*),...,*e*_*p*_(*i*))^*T *^(*i *= 1,...,*k*), we have

(1) $$S=\sum_{i,j}w_{ij}(c_{i}^{*}-c_{j}^{*}{)}^{2}$$

(2) $$=(f_{1},...,f_{p})E_{lab}^{T}\cdot L\cdot E_{lab}{\left(f_{1},...,f_{p}\right)}^{T}$$

(3) $$=\sum_{i=1}^{p}\lambda_{i}f_{i}^{2}$$

Applying SVM with the linear kernel to *E*_*lab *_is essentially to perform a linear operation on *E*_*lab*_, such as $(f_{1},...,f_{p})E_{lab}^{T}$. Then  can be naturally considered as the classifiers, and so equation (1) measures the weighted differences among the objects' classification labels with *w*_*ij*_'s being the weights. We note that the labelling differences associated with larger *w*_*ij*_'s (corresponding to the pairs of objects with higher similarities) are having more weights. Hence, the smaller *S *is, the more likely that the objects with high similarities would have the same class label. Furthermore, from equation (3), our manifold data naturally leads to the smallest S for a given linear operation and *p *due to the use of the smallest eigenvalues/eigenvectors. In brief, the manifold transformation helps better distinguish between the positive and negative sets and thus further improves the classification results.

## List of abbreviations used

C-SVC: C-support vector classification; FIM: Frequent Itemset Mining; GEO: Gene Expression Omnibus; LOOCV: Leave One dataset Out Cross Validation; MeSH: Medical Subject Heading; SVD: Singular Value Decomposition; SVM: Support Vector Machine; tf-idf: term frequency-inverse document frequency; UMLS: Unified Medical Language Systems.

## Competing interests

The authors declare that they have no competing interests.

## Authors' contributions

CCL, JH, HH, and XJZ designed the study. CCL, JH, and MK performed the study. CCL, HH, and XJZ analyzed the result. CCL, HH, and XJZ wrote the paper.
